# Low-Carbon and Nanosheathed ZnCo_2_O_4_ Spheroids with Porous Architecture for Boosted Lithium Storage Properties

**DOI:** 10.34133/2019/1354829

**Published:** 2019-08-21

**Authors:** Yudi Mo, Junchen Liu, Shuanjin Wang, Min Xiao, Shan Ren, Dongmei Han, Yuezhong Meng

**Affiliations:** ^1^The Key Laboratory of Low-Carbon Chemistry & Energy Conservation of Guangdong Province, State Key Laboratory of Optoelectronic Materials and Technologies, Sun Yat-sen University, Guangzhou 510275, China; ^2^School of Chemical Engineering and Technology, Sun Yat-sen University, Guangzhou 510275, China

## Abstract

Multielectronic reaction electrode materials for high energy density lithium-ion batteries (LIBs) are severely hindered by their inherent sluggish kinetics and large volume variations, leading to rapid capacity fade. Here, a simple method is developed to construct low-carbon and nanosheathed ZnCo_2_O_4_ porous spheroids (ZCO@C-5). In this micro/nanostructure, an ultrathin amorphous carbon layer (~2 nm in thickness) is distributed all over the primary nanosized ZCO particles (~20 nm in diameter), which finally self-assembles into porous core (ZCO)-shell(carbon) micron spheroids. The nanoencapsulation and macro/mesoporous architecture can not only provide facile electrolyte penetration and rapid ion/electron transfer but also better alleviate volumetric expansion effect to avoid pulverization of ZCO@C-5 spheroids during repeat charge/discharge processes. As expected, the three-dimensional porous ZCO@C-5 composites exhibit high reversible capacity of 1240 mAh g^−1^ cycle at 500 mA g^−1^, as well as excellent long-term cycling stability and rate capability. The low-carbon and nanoencapsulation strategy in this study is simple and effective, exhibiting great potential for high-performance LIBs.

## 1. Introduction

Currently, lithium-ion batteries (LIBs) as important energy storage devices are widely applied in various fields, such as portable electronic devices and electric vehicles, and so on [1–3]. The insertion-type Li_4_Ti_5_O_12_ and carbon materials have been widely applied as commercial anode for LIBs. This type of material owns small volume change and stable cycling performances during repeat Li^+^ insertion/extraction processes, while their low storage capacity has become one of the main obstacles for the further development [4, 5]. Therefore, it is necessary to exploit alternative anode materials with high energy density, long cycling stability, and low cost for high-performance LIBs [6, 7].

Transition metal oxides (TMOs), particularly cobalt (II, III) oxide (Co_3_O_4_), have been extensively investigated as promising anode materials for LIBs, which can offer higher active capacity (890 mAh g^−1^) than that of insertion-type anodes [8–11]. However, the high-cost and great toxicity of Co restrict their practical applications. Thus, substituting Co by much cheaper and ecofriendly elements (Fe, Zn, Mn, and Ni, etc.) is an effective solution [12–16]. Among them, spinel ZnCo_2_O_4_ (ZCO) has received widespread attention in the past decades. At first, the introduction of Zn element can perform a synergistic effect between bimetallic elements (Zn and Co), which would improve electrical conductivity, crystal structure stability, and electrochemical activity of composites [15]. Besides, in addition to conversion reaction (Zn^2+^ + 2e^−^*↔* Zn) during charge storage, the alloying reaction can also be performed between Zn and Li (Zn + Li^+^ + e^−^*↔* LiZn) [17, 18]. As a result, it can produce a very high reversible capacity. However, different from the insertion-type anode materials, low conductivity, huge volume expansion/contraction effect, and severe self-aggregation of spinel ZCO during lithiation/delithiation cycling will lead to unsatisfactory electrochemical performances [19]. Therefore, a variety of nanostructures for ZCO have been reasonably designed toward overcoming the above issues, such as 2D ultrathin ZCO nanosheets [20], orange-shaped ZCO [21], hollow ZCO nanocages [13], ZCO-urchins-on-carbon-fibers [22], RGO@ZCO quantum dots [23], ZCO@CNTs,[24]. Moreover, the past decade has witnessed that the loose structure design could effectively alleviate volumetric expansion effect, and the introduction of carbon (CNTs, rGO, etc.) would further improve the electrical conductivity and structure stability of the active materials [25–27]. Carbon materials, just like a golden key, play a vital role in the preparation of electrode materials [28–31]. In addition, the coupling effects between carbon and TMOs may bring additional properties to improve the performance, for instance, stability and initial coulombic efficiency [32, 33] However, it remains as a great challenge to integrate all these features of hybrid structures. Compared to the common CNTs and rGO, organic carbon source has its unique advantages to achieve uniform coating of nanomaterials with various architectures and control thickness of the coating layer, which will lead to satisfactory electrochemical performances.

**Scheme 1:**
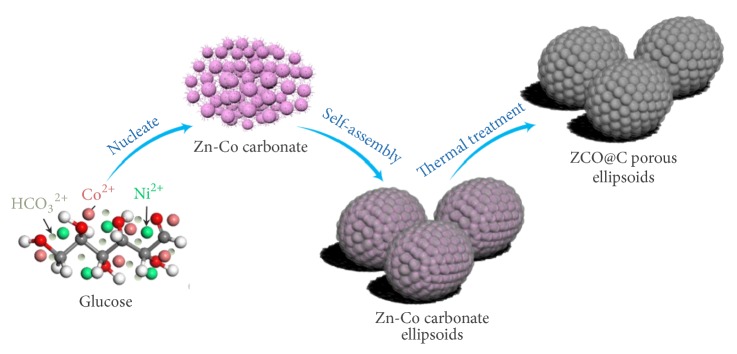
Schematic illustration for the synthesis process of micro/nano ZCO@C-5 porous spheroids.

Along this line, we develop a facile route to fabricate low-carbon and nanoencapsulation ZCO porous spheroids (ZCO@C-5) via hydrothermal reaction combined with subsequent high-temperature treatment, as shown in Scheme 1. In the process of hydrothermal reaction, as a slowly released pH-adjusting agent, the generating OH^−^ ions from urea prompt the hydrolysis of HCO_3_^−^. Then, hydrolyzed CO_3_^2−^ anions will react with metal ions (Zn^2+^ and Co^2+^) to form Zn-Co carbonate nanoparticles. Based on the certain chelation of oxygen-containing groups (-OH and/or -COOH) to metal ions, the glucose molecules also play an important role in coordinating crystal growth and binding individual Zn-Co carbonate nanoparticles during the self-assembly process of precursor [34]. Meanwhile, the gradual carbonization of glucose will form uniform coating layer to encapsulate Zn-Co carbonate nanoparticles under the hydrothermal condition. Finally, the porous ZCO@C-5 spheroids can be obtained with well-maintained morphology via a facile high-temperature heat treatment. The results revealed that the design of such a porous core(ZCO)-shell(carbon) micro/nanostructure can provide rapid ion/electron transport pathways and enhance the structural stability of porous spheroids, which will effectively solve the pulverization problem of electrode materials during the repeat charge/discharge progress. Besides, the forming ultrathin carbon layer will not significantly hinder Li^+^ transport from electrolyte into ZCO phase. Herein, the high electrochemical performance can be afforded.

## 2. Results

### 2.1. Phase and Microstructures of Synthesized Materials

**Figure 1:**
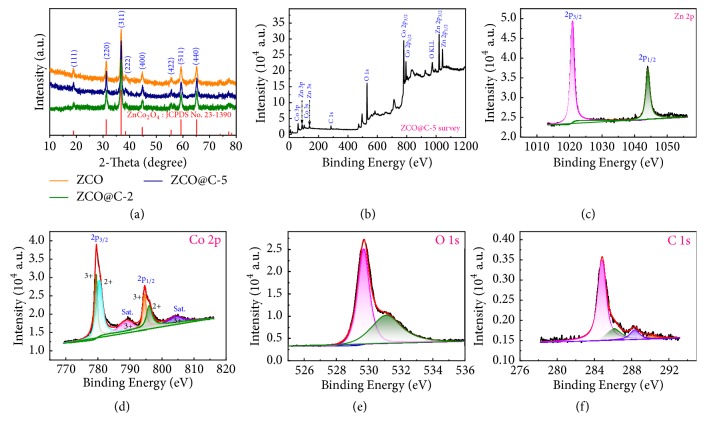
(a) XRD pattern of ZCO, ZCO@C-2, and ZCO@C-5 samples; XPS spectra of ZCO@C-5 composites: (b) survey spectrum, (c) Zn 2p, (d) Co 2p, (e) O 1s, and (f) C 1s.

The crystal structure and chemical composition of materials were characterized by XRD, XPS, FTIR, Raman, and TG tests (Figure 1; Figures [Supplementary-material supplementary-material-1]-[Supplementary-material supplementary-material-1], Supplementary Materials). As shown in [Supplementary-material supplementary-material-1], the precursors of ZCO, ZCO@C-2, ZCO@C-5, and ZCO@C-10 (the samples with addition of 0 g, 2 g, 5 g, and 10 g of glucose) are identified as Zn-Co carbonate, which is confirmed by that ZnCO_3_ (JCPDS card No. 83-1765) and CoCO_3_ (JCPDS card No. 78-0209) peaks can be observed in XRD patterns. It is well known that the forming carbon of hydrothermal carbonation of glucose is amorphous. So, there are no other carbon peaks observed in XRD patterns. After being calcined at 400°C for 5 h, all the diffraction peaks of ZCO, ZCO@C-2, and ZCO@C-5 can be indexed to the cubic ZnCo_2_O_4_ (JCPDS No. 23-1390, a=b=c=8.0946 Å) in Figure 1(a). No additional diffraction peaks are found, indicating the complete conversion of Zn-Co carbonate precursors to cubic ZCO. However, the ZCO@C-10 composites exhibit entirely different XRD diffraction peaks ([Supplementary-material supplementary-material-1]), which are in good consistency with hexagonal ZnO (JCPDS card No. 89-0510) and cubic CoO (JCPDS card No. 78-0431). The addition of excess glucose will result in the transformation of the crystal structure during the final high-temperature calcination process and form mixed metal oxide. The FTIR spectrum of samples is shown in [Supplementary-material supplementary-material-1]. Two obvious absorption peaks located at 574 cm^−1^ and 775 cm^−1^ for ZCO, ZCO@C-2, and ZCO@C-5 are assigned to metal-O bonds [35]. For ZCO@C-10, an obviously different absorption peak between 500 cm^−1^ and 900 cm^−1^ implies the structure discrepancy of the prepared sample, which has been confirmed by XRD patterns (Figure 1(a), [Supplementary-material supplementary-material-1]). In addition, we also notice that the signal from carbon can be detected in XPS (Figure 1(b)) and Raman spectra ([Supplementary-material supplementary-material-1]), which further proves that the formed carbon is amorphous. From the XRD pattern of carbon in [Supplementary-material supplementary-material-1], the two wide peaks of amorphous carbon derived from D-glucose treated by hydrothermal reaction and high-temperature calcination can be detected. The carbon contents of ZCO@C-2, ZCO@C-5, and ZCO@C-10 composites estimated by TG analysis are about 0.92, 1.39, and 19.89 wt.% ([Supplementary-material supplementary-material-1]).

The XPS technique was employed to further study the element compositions and the chemical states of ZCO@C-5 composites. Figure 1(b) shows the typical full spectrum of ZCO@C-5 composites, indicating the existence of Zn, Co, O, and C elements. In the high-resolution XPS spectrum of Zn 2p (Figure 1(c)), two representative peaks located at 1020.9 eV for Zn 2p_3/2_ and 1044.0 eV for Zn 2p_1/2_ reveal the characteristic of Zn^2+^. In Figure 1(d), the Co 2p spectrum was also well-fitted by the Gaussian fitted method. Two kinds of Co species (Co^2+^ and Co^3+^ ions) and two shakeup satellites (denoted as “Sat.”) can also be observed. As shown in Figure 1(e), the O 1s spectrum can be divided into two major peaks. The fitting peak centered at 529.7 eV is typical of oxygen species in ZnCo_2_O_4_. Besides, the fitting peak at 531.1 eV corresponds to the adsorbed oxygen and hydroxyl group. The C 1s spectrum (Figure 1(f)) exhibits three fitting peaks located at 284.8 eV (C-C bonds), 286.1 eV (O-C-O complex), and 288.3 eV (carbonate species). The above results are well consistent with the previous reports [13, 36–38].

**Figure 2:**
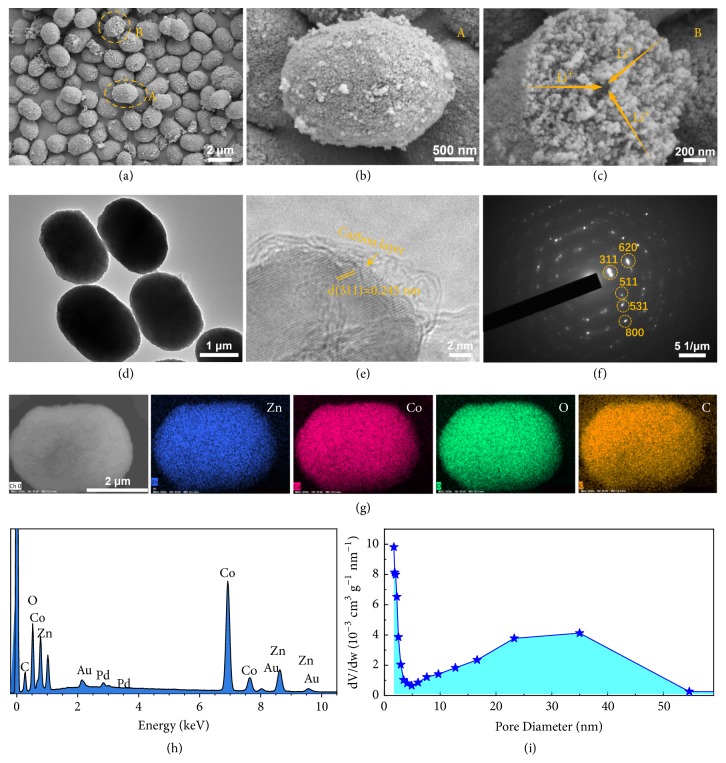
SEM images(a-c), TEM images (d, e), selected area electron diffraction (SAED) pattern (f), EDS mapping (g) with the corresponding EDS report (h), and pore size distribution (i) of as-synthesized ZCO@C-5 porous ellipsoids.

The detailed morphology and microstructure of micro/nano ZCO@C-5 porous ellipsoids were examined by SEM and TEM. As shown in [Supplementary-material supplementary-material-1], the Zn-Co carbonate ellipsoids are uniform with relatively smooth surface. Upon thermal treatment, the ZCO@C-5 composites can still preserve original morphology of precursor (Figures 2(a) and 2(b)). But its surface became quite rough because the thermal decomposition of carbonate resulted in porous structure. A further observation reveals that each ellipsoid consists of numerous smaller nanoparticles (~20 nm), finally forming into a dense ellipsoid. From a broken ellipsoid (Figure 2(c)), it can be clearly observed that the interior of ZCO@C-5 composites is still loose and porous, which can provide abundant electrode-electrolyte interfaces for Li^+^ storage and realize fast ions transfer. The morphological changes of samples with different amounts of glucose were also studied ([Supplementary-material supplementary-material-1]). Without the introduction of D-glucose, the pure ZCO will grow bigger with a diameter of approximately 9.2 *μ*m (Figures [Supplementary-material supplementary-material-1] and [Supplementary-material supplementary-material-1]). However, a small amount of D-glucose (2 g) will not cause a significant morphology change in particle size of ZCO@C-2 (~8.8 *μ*m, Figures [Supplementary-material supplementary-material-1] and [Supplementary-material supplementary-material-1]). When the D-glucose additive amount is five times as high as that of ZCO@C-2, the obtained ZCO@C-10 particles become smaller and irregular (Figures [Supplementary-material supplementary-material-1], [Supplementary-material supplementary-material-1]), indicating that the excess glucose will seriously affect the self-assembly process of Zn-Co carbonate crystal due to the abundant oxygen-containing groups and the formation of the massive amorphous carbon in the hydrothermal process.

Figure 2(d) shows the TEM images of intact ZCO@C-5 ellipsoids with a length of ~2.1 *μ*m and a width of ~1.5 *μ*m. The enlarged TEM images (Figure 2(e)) exhibit one selected edge area of ZCO@C-5 composites. The surface of ZCO@C-5 primary particles is coated by a layer of ultrathin amorphous carbon (~2 nm in thickness) derived from hydrothermal carbonization of D-glucose. Meanwhile, the inside well-defined lattice fringes with a space of 0.245 nm can be indexed as [311] lattice plane of ZCO crystal. In addition, the discontinuous diffraction rings shown in SAED pattern (Figure 2(f)) indicate the polycrystalline nature, and these rings can be satisfactorily indexed to the cubic ZCO phase. The uniform distribution of Zn (blue), Co (pink), O (green), and C (orange) elements in the ZCO@C-5 ellipsoids is demonstrated by EDS maps in Figure 2(g), which further reveals that the ZCO ellipsoids can be well coated by carbon layer. The quantitative analysis by ICP-MS further confirms that the Co/Zn atomic ratio in the samples is about 2.3 ([Supplementary-material supplementary-material-1]). The pore size distribution data in Figure 2(i) show that the pore sizes of ZCO@C-5 porous ellipsoids are in the range of 1.2~60 nm. And all the N_2_ adsorption isotherm curves of samples in [Supplementary-material supplementary-material-1] are identified as typical type IV with a distinct H3-type hysteresis loops, which can be linked to slit-shaped pores. BET specific surface areas of ZCO, ZCO@C-2, ZCO@C-5, and ZCO@C-10 are 35.82, 35.93, 43.74, and 19.61 m^2^ g^−1^, respectively. The rich porous texture and larger specific surface of ZCO@C-5 composites come from its reasonable structure construction, which possibly promotes a considerable improvement in the electrochemical properties.

### 2.2. Electrochemical Analysis

**Figure 3:**
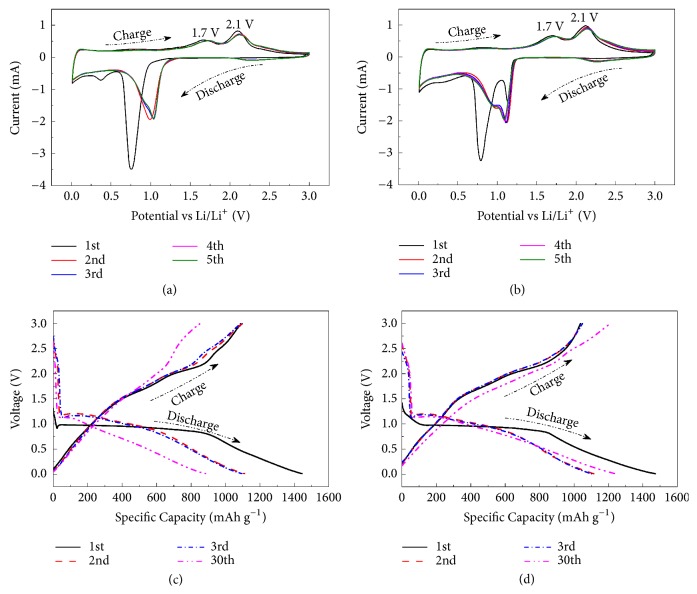
CV curves of pure ZCO (a) and ZCO@C-5 composites (b) at 0.2 mV s^−1^ with a cut-off voltage range of 0.01-3.0 V. Discharge-charge profiles of pure ZCO (c) and ZCO@C-5 composites (d) in the 1st, 2nd, 3rd, and 30th cycles at 500 mA g^−1^.

Firstly, the electrochemical performances of pure ZCO and ZCO@C-5 composites as anodes for LIBs were evaluated by CV and galvanostatic discharge-charge tests. The representative CV curves for ZCO and ZCO@C-5 electrodes are shown in Figures 3(a) and 3(b). Two distinct cathodic peaks can be observed in the first cycle between 0.5 and 1.5 V, which derive from reduction process of ZnCo_2_O_4_ to Zn^0^ and Co^0^ accompanied by the formation of solid electrolyte interphase (SEI) layers during lithiation process (Eqn. (1)) [39]. The weak and broad peak centered at 0.3 V results from alloying reaction between Li^+^ and Zn (Eqn. (2)) [40, 41].

(1) $$\begin{array}{c} ZnCo_{2}O_{4}+8{Li}^{+}+8e^{-}\overset{1st\;\;discharge}{\to } \end{array}$$

(2) $$\begin{array}{c} {Li}^{+}+Zn+e^{-}\overset{DisCharge}{\underset{Charge}{⇄}}LiZn \end{array}$$

Different from that of the first cathodic curve, other cathodic peaks become weak and shift to 0.95 and 1.1 V corresponding to the reduction of ZnO to metallic Zn (Eqn. (3)) and Co_3_O_4_ to metallic Co (Eqn. (4) and (5)) in the subsequent cycles, which reflects the irreversible electrochemical reaction during the first discharge cycle in Eqn. (1). Individual ZnO and Co_3_O_4_ as anode material for LIBs have been clearly reported in many previous works [8, 9, 42, 43].

(3) $$\begin{array}{c} Zn+{Li}_{2}O\overset{Charge}{\underset{Discharge}{⇄}}\\ ZnO+2{Li}^{+}+2e^{-} \end{array}$$

(4) $$\begin{array}{c} 2Co+2{Li}_{2}O\overset{Charge}{\underset{Discharge}{⇄}}\\ 2CoO+4{Li}^{+}+4e^{-}\\ \frac{2}{3}{Co}_{3}O_{4}+\frac{4}{3}{Li}^{+}+\frac{4}{3}e^{-} \end{array}$$

(5) $$\begin{array}{c} 2CoO+\frac{2}{3}{Li}_{2}O\overset{Charge}{\underset{Discharge}{⇄}}\\ \frac{2}{3}{Co}_{3}O_{4}+\frac{4}{3}{Li}^{+}+\frac{4}{3}e^{-} \end{array}$$

In all anodic processes, two main oxidation peaks at 1.7 and 2.1 V are attributed to the reoxidation of Zn and Co (Eqn. (3)-(5)), respectively [41]. After the first cycle, the CV profiles overlap very well, indicating good electrochemical reversibility of ZCO@C-5 composites for Li^+^ insertion/extraction.

Typical discharge and charge characteristics for the 1st, 2nd, 3rd, and 30th cycles of pure ZCO and ZCO@C-5 composites at 500 mA g^−1^ are shown in Figures 3(c) and 3(d). In the first discharge curve, both electrodes exhibit an initial sudden voltage drop up to 1.3 V, a long voltage plateau at ~0.97 V and a slow ramp voltage to 0.01 V, which are in accordance the CV results. The initial fast voltage drop implies that there are almost no electrochemical reaction processes at this voltage range. Especially, the discharge voltage plateau of the first cycle is different from the following cycles, which is related to the irreversible reactions of SEI and phase separation of Zn and Co oxides during the first cycle. The initial discharge/charge specific capacities of ZCO@C-5 electrode are 1477/1037 mAh g^−1^ with 70.2% coulombic efficiency, whereas 1443/1097 mAh g^−1^ for pure ZCO with 76.0% coulombic efficiency. For both electrodes, the irreversible capacity loss in the initial cycle derives from the irreversible electrochemical reactions involved in the formation of SEI films and the decomposition of electrolyte, which also occur in the most anode materials [7, 44, 45].

**Figure 4:**
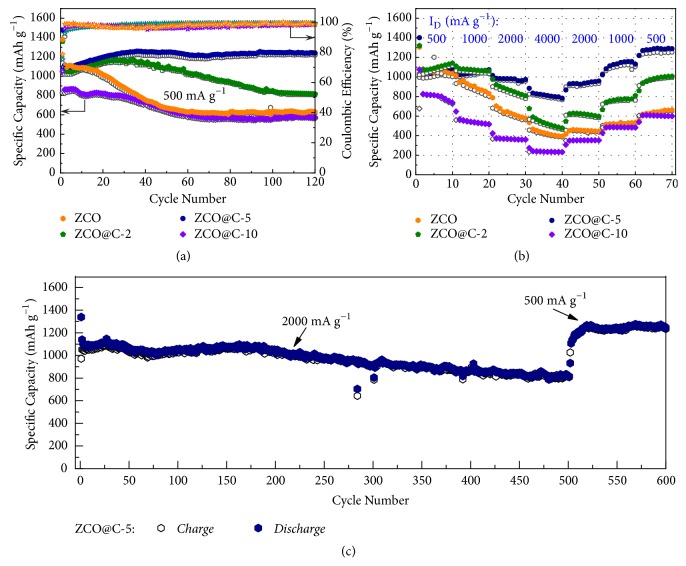
(a) Cycling performances of samples at 500 mA g^−1^ with the corresponding coulombic efficiency; (b) rate capabilities of samples at various current densities from 500 to 4000 mA g^−1^; (c) long-term cycling performance of ZCO@C-5 composites under large current density.

To investigate the potential application of the ZCO@C-5 composites as high-performance anode, the comparative cycling performances and rate capabilities of ZCO, ZCO@C-2, ZCO@C-5, and ZCO@C-10 samples are shown in Figure 4. Apparently, ZCO@C-5 composites demonstrate much better cyclic capacity retention than other electrode materials (Figure 4(a)). When tested at 500 mA g^−1^, the ZCO@C-5 electrode exhibits a gradually increasing reversible capacity from 2nd cycle to 30th cycle, ascribed to an activation process of electrode material during cycling [46, 47]. Then the ZCO@C-5 electrode maintains a stable trend and finally retains a high discharge capacity of 1240 mAh g^−1^ after 120 cycles. However, the other three electrode materials suffer different degrees of capacity decay. After 120 cycles, the capacity retention ratios (versus the second discharge capacity) of ZCO, ZCO@C-2, and ZCO@C-10 are about 56.8%, 75.2%, and 66.9%, respectively, confirming the forming amorphous carbon coating layer can improve the cycling stability of electrode materials. In addition, the lower reversible capacity of ZCO@C-10 can be ascribed to its high carbon content (19.89 wt.%).

As expected, the ZCO@C-5 composites also show excellent rate capability (Figure 4(b)). At elevated current densities, the ZCO@C-5 electrode delivers remarkable reversible capacities of 1051, 1048, 981, and 818 mAh g^−1^ at 500, 1000, 2000, and 4000 mA g^−1^, respectively. When the current density back to 500 mA g^−1^ for another 10 cycles, a high reversible capacity of 1278 mAh g^−1^ can be recovered. These results suggest that the micro/nanoarchitecture of ZCO@C-5 porous ellipsoids is quite stable and not damaged under a large current. In contrast, ZCO, ZCO@C-2, and ZCO@C-10 electrodes undergo a rapid capacity fading with increase of current density and only deliver a low reversible capacity of 435, 540, and 239 mAh g^−1^ at 4000 mA g^−1^, in turns. Besides, the discharge-charge voltage profiles of pure ZCO and ZCO@C-5 composites depending on various current densities are shown in [Supplementary-material supplementary-material-1]. It can be seen that the ZCO@C-5 electrode exhibits a higher discharge voltage and a lower charge voltage at each rate than that of pure ZCO electrode, revealing its lower electrode polarization.

Besides, high-rate cycling performance is also an important parameter for LIBs in practical application because it can shorten charging time and satisfy high-power applications. Therefore, the ZCO@C-5 electrode was also evaluated at a higher current density of 2000 mA g^−1^ in Figure 4(c). After 500 cycles, it still delivers a high reversible capacity of 815 mAh g^−1^. With the current density back to 500 mA g^−1^ for the subsequent 100 cycles, the high reversible capacity of 1243 mAh g^−1^ can be recovered at 600th cycle, demonstrating the excellent cycling performance.

**Figure 5:**
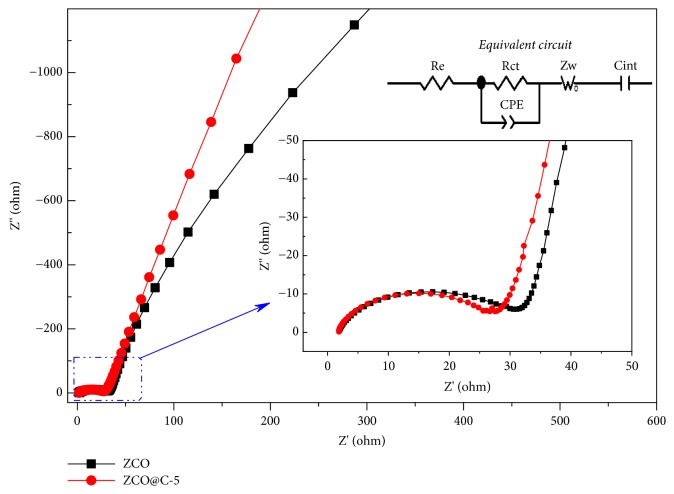
EIS spectra of pure ZCO and ZCO@C-5 composites.

The kinetic properties of pure ZCO and ZCO@C-5 composites were studied by EIS measurements in Figure 5. The equivalent electrical circuit is shown in Figure 5 (inset), in which* CPE* and* C*_*int*_ are double-layer capacitance and intercalation capacitance,* R*_*ct*_ and* R*_*e*_ present interfacial charge transfer resistance and electrolyte resistance, and* Z*_*w*_ is associated with Warburg impedance of Li^+^ diffusion into ZCO crystal [39, 46]. The corresponding fitting results are listed in [Supplementary-material supplementary-material-1]. The* R*_*ct*_ value of ZCO@C-5 electrode is smaller than that of pure ZCO electrode, indicating its lower interfacial charge transfer resistance. The introduced conductive carbon networks not only improve the kinetics of electron transport, but also strengthen the structural stability of active materials. Therefore, the ZCO@C-5 electrode exhibits excellent electrochemical behaviors. As shown in [Supplementary-material supplementary-material-1], the cycling performance and rate capability of our designed micro/nano ZCO@C-5 porous architecture are comparable or superior to those of many previously reported representative electrode materials. The excellent electrochemical performances of ZCO@C-5 half cell urge us to further evaluate its practicability in full cells, as shown in [Supplementary-material supplementary-material-1]. Based on the mass of ZCO@C-5 composites, the LMO/ZCO@C-5 full cell exhibits a high discharge capacity of 565 mAh g^−1^ after 20 cycles at 100 mA g^−1^, and the corresponding average output voltage is as high as 2.3 V, which indicates its great potential application.

**Figure 6:**
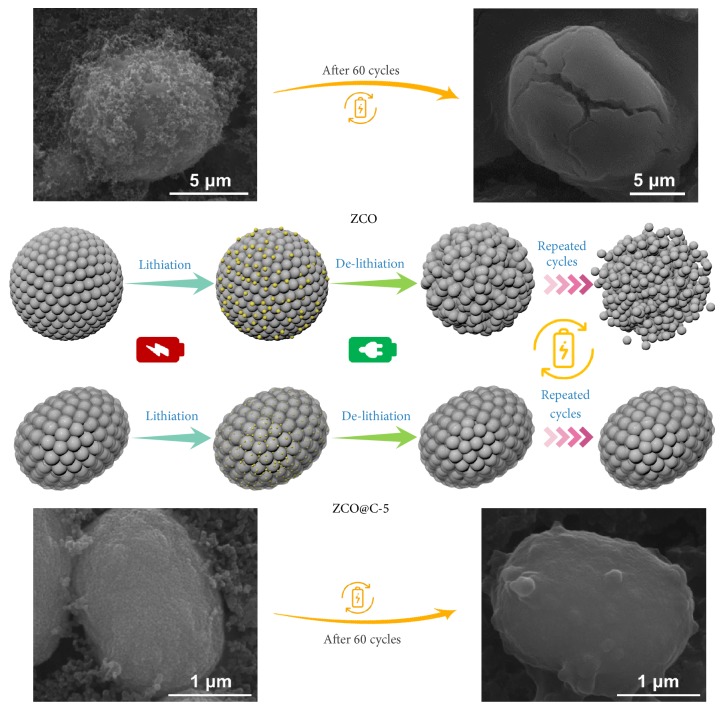
SEM images and corresponding morphology evolution of pure ZCO and ZCO@C-5 composites during discharge/charge processes.

Figure 6 illustrates the morphology evolution of pure ZCO and ZCO@C-5 composites during discharge/charge cycling. Due to large volume fluctuations during the repeated conversion reaction, the architecture of pure ZCO can be easily broken and thus lead to cause electrode pulverization and capacity loss. To verify the structural stability of low-carbon and nanosheathed ZCO@C-5 porous spheroids, pure ZCO and ZCO@C-5 composites after 60 cycles were observed by SEM. As shown in Figure 6, in contrast to the severe structural damage observed in the SEM images of pure ZCO, the structure of ZCO@C-5 spheroids can be well maintained, confirming its good structure stability.

In brief, the high reversible capacity, enhanced rate capability, and long-term cycling stability of the ZCO@C-5 composites can be attributed to the following aspects: firstly, lots of macro/mesoporous of ZCO@C-5 ellipsoids increase electrode-electrolyte contact area in order to permit more Li^+^ ions into the inner region. Secondly, the nanosized ZCO particles (~20 nm) shorten the diffusion distance of Li^+^ ions in ZCO crystal, which is very conducive to the full play of the active capacity. Thirdly, massive interstitial space among primary particles will provide enough buffer area and effectively relax internal strain. Fourthly, the forming carbon conductive network can improve electrical conductivity and inhibit the aggregation of active materials during electrochemical reaction process. Fifthly, the outmost carbon layer as a structural reinforcement can maintain the integrity of porous ZCO@C-5 ellipsoids to overcome electrode pulverization during repeat charge/discharge process.

## 3. Conclusions

In summary, the novel micro/nano ZCO@C-5 ellipsoids with abundant porous characteristic and ultra-low carbon content were successfully prepared through facile solvothermal method and subsequent high-temperature heat treatment. Benefiting from its convenient ion transport, fast charge transfer, and excellent structural stability, the obtained carbon-sheathed ZCO@C-5 porous ellipsoids with 1.39 wt.% carbon content exhibit high reversible capacity of 1240 mAh g^−1^ and excellent large-rate cyclability with a capacity of 815 mAh g^−1^ at 2000 mA g^−1^ after 500 cycles. It can be concluded that this strategy is simple and effective, which can be used for fast synthesis of carbon-encapsulated micro/nano porous composites for energy storage devices and other research fields.

## 4. Methods

### 4.1. Synthesis of 3D Porous ZCO@C-5 Nanoarchitectures

The 3D micro/nano ZCO@C-5 porous spheroids were synthesized by a facile two-step method. Firstly, Zn(NO_3_)_2_·6H_2_O (4 mmol), Co(NO_3_)_2_·6H_2_O (8 mmol), urea (20 mmol), and D-glucose (5 g) were in turn added to a mixture solution of 20 mL deionized water and 80 mL ethylene glycol. The mixture was stirred for 12 h to await chelation. Subsequently, NH_4_HCO_3_ (120 mmol) was quickly added to the above homogeneous solution. After continuously stirring for another 0.5 h, the prepared reaction solution was sealed into a 150 mL Teflon vessel and heated to 200°C for 20 h. After natural cooling, the black precipitate was washed and dried at 80°C overnight. To obtain porous ZCO@C-5 composites, the above precursor was thermally treated at 400°C for 5 h.

For comparison, by changing the additive amount of D-glucose to 0 g, 2 g, and 10 g, the prepared samples were marked as ZCO, ZCO@C-2, and ZCO@C-10, and the using reagents and processing methods were the same as above mentions.

### 4.2. Material Characterization

Powder X-ray diffraction (XRD; Hitachi Rigaku/D-MAX 2200 VPC, Cu/K*α* radiation, *λ*=1.5406 Å) pattern was carried out to collect the crystallographic information of as-prepared materials. Thermogravimetric analysis (PerkinElmer SII) was conducted under air flow to explore thermodynamic properties and carbon content of samples. Scanning electron microscopy (SEM; ZEISS ULTRA 55) and transmission electron microscopy (TEM; JEOL, JEM-2100HR) with energy-dispersive X-ray spectroscopy (EDS) were performed to obtained the morphology and microstructure information. The element valences of the samples were characterized by X-ray photoelectron spectroscopy (XPS, ESCALab250). The Raman spectra were obtained by Raman spectrometer (HR800UV) to study the order/disorder crystal structures of ZCO@C-5 composites. Brunauer-Emmett-Teller (BET; ASAP2460 analyzer) and Barret-Joyner-Halenda (BJH) methods were employed to characterize specific surface area and pore size distribution, respectively. ICP-MS analysis was performed to obtain the metal element content of the samples by a Perkin Elmer Optima 8300.

### 4.3. Cell preparation and Electrochemical Analysis

The working electrodes were prepared by mixing 80 wt.% active materials (pure ZCO, ZCO@C-2, ZCO@C-5, or ZCO@C-10), 10wt.% conductive agent (Super P), 10 wt.% binder (LA133) and proper amount of DI water to form about 38 wt.% homogeneous slurry. The electrode slurry was coated onto the conductive current collector (Cu foil). The electrodes were dried at 60°C in oven overnight. The electrode density of active materials was 1.1-1.4 mg cm^−2^. The electrochemical performances were evaluated by coin-type cells (CR2025) with lithium metal as the reference electrode, and Celgard 2500 membrane as separator. The electrolyte was 1.0 M LiPF_6_ in a mixture of ethylene carbonate, dimethyl carbonate, diethyl carbonate (1:1:1 by volume). The prepared working electrodes have a very good wettability with electrolyte ([Supplementary-material supplementary-material-1]). Finally, all the coin-type cells were assembled in an Ar-filled glovebox.

Cyclic voltammetry (CV, 0.01-3.0 V) and electrochemical impedance spectroscopy (EIS, 10 mHz-100 KHz) were performed on CHI604E electrochemical workstation. Galvanostatic and rate charge/discharge performances were tested by a LAND system with a cut-off voltage range of 0.01-3.0 V.

The full-cells were fabricated with the commercial LiMn_2_O_4_ (LMO) as the cathode and the prepared ZCO@C-5 electrode as the anode. The full-cells were tested between 1.0 V and 3.6 V. The other conditions are the same as the half cells.

## Data Availability

All data analyzed during this study are present in the paper and the Supplementary Materials.

## Supplementary Materials

Supplementary Materials Figure S1: XRD patterns of the as-prepared precursors of ZCO, ZCO@C-2, ZCO@C-5, and ZCO@C-10. Figure S2: XRD pattern of the ZCO@C-10. Figure S3: FTIR spectra of ZCO, ZCO@C-2, ZCO@C-5, and ZCO@C-10. Figure S4: Raman pattern of ZCO@C-5 composite. Figure S5: XRD pattern of carbon derived from pure D-glucose treated by hydrothermal reaction and high-temperature calcination. Figure S6: TG curve of the pure Zn-Co carbonate precursor. Figure S7: TG curves of the as-prepared (a) ZCO@C-2, (b) ZCO@C-5, and (c) ZCO@C-10 composites. Figure S8: SEM images of ZCO@C-5 precursor. Figure S9: SEM and TEM images of (a-c) pure ZCO, (d-f) ZCO@C-2, and (g-i) ZCO@C-10 samples. Figure S10: nitrogen adsorption-desorption isotherm of porous ZCO@C-5 composites (a). Nitrogen adsorption-desorption isotherm and corresponding pore size distribution (inset) of (b) ZCO, (c) ZCO@C-2, and (d) ZCO@C-10. Figure S11: discharge-charge voltage profiles of pure ZCO and ZCO@C-5 composites at various current densities: 500 mA g^−1^, 1000 mA g^−1^, 2000 mA g^−1^, and 4000 mA g^−1^. Figure S12: (a) discharge-charge voltage profiles and (b) cycling performance at 100 mA g^−1^ with corresponding coulombic efficiency of the LMO/ZCO@C-5 full cell. Figure S13: SEM images of ZCO@C-5 electrode after 600 cycles. Table S1: Zn and Co content of ZCO and ZCO@C-5 samples determined by ICP-MS. Table S2: kinetic parameters of pure ZCO electrode and ZCO@C-5 composites electrode. Table S3: performance comparison between our materials with representative anode materials.
