# Supplementary material for: Low-Carbon and Nanosheathed ZnCo2O4 Spheroids with Porous Architecture for Boosted Lithium Storage Properties
Source: Research (Wash D C). 2019 Aug 21;2019:1354829. doi: 10.34133/2019/1354829 (PMC6753607; doi:10.34133/2019/1354829)
Supplement: Supplementary Materials — Figure S1: XRD patterns of the as-prepared precursors of ZCO, ZCO@C-2, ZCO@C-5, and ZCO@C-10. Figure S2: XRD pattern of the ZCO@C-10. Figure S3: FTIR spectra of ZCO, ZCO@C-2, ZCO@C-5, and ZCO@C-10. Figure S4: Raman pattern of ZCO@C-5 composite. Figure S5: XRD pattern of carbon derived from pure D-glucose treated by hydrothermal reaction and high-temperature calcination. Figure S6: TG curve of the pure Zn-Co carbonate precursor. Figure S7: TG curves of the as-prepared (a) ZCO@C-2, (b) ZCO@C-5, and (c) ZCO@C-10 composites. Figure S8: SEM images of ZCO@C-5 precursor. Figure S9: SEM and TEM images of (a-c) pure ZCO, (d-f) ZCO@C-2, and (g-i) ZCO@C-10 samples. Figure S10: nitrogen adsorption-desorption isotherm of porous ZCO@C-5 composites (a). Nitrogen adsorption-desorption isotherm and corresponding pore size distribution (inset) of (b) ZCO, (c) ZCO@C-2, and (d) ZCO@C-10. Figure S11: discharge-charge voltage profiles of pure ZCO and ZCO@C-5 composites at various current densities: 500 mA g−1, 1000 mA g−1, 2000 mA g−1, and 4000 mA g−1. Figure S12: (a) discharge-charge voltage profiles and (b) cycling performance at 100 mA g−1 with corresponding coulombic efficiency of the LMO/ZCO@C-5 full cell. Figure S13: SEM images of ZCO@C-5 electrode after 600 cycles. Table S1: Zn and Co content of ZCO and ZCO@C-5 samples determined by ICP-MS. Table S2: kinetic parameters of pure ZCO electrode and ZCO@C-5 composites electrode. Table S3: performance comparison between our materials with representative anode materials. [file 1354829.f1.docx]

Supplementary Materials

**Low-Carbon and Nano-Sheathed ZnCo_2_O_4_ Spheroids with Porous Architecture for Boosted Lithium Storage Properties**

Yudi Mo,^1^ Junchen Liu,^1^ Shuanjin Wang,^1^ Min Xiao,^1^ Shan Ren,^1^ Dongmei Han,^2,*^ and Yuezhong Meng^1,*^

^1^ *The Key Laboratory of Low-carbon Chemistry & Energy Conservation of Guangdong Province, State Key Laboratory of* Optoelectronic *Materials and Technologies, Sun Yat-sen University, Guangzhou 510275, PR China.*

*^2^* *School of Chemical Engineering and Technology, Sun Yat-sen University, Guangzhou 510275, PR China.*

Correspondence should be addressed to Dongmei Han; [handongm@mail.sysu.edu.cn](mailto:handongm@mail.sysu.edu.cn) and Yuezhong Meng; [mengyzh@mail.sysu.edu.cn](mailto:mengyzh@mail.sysu.edu.cn)

**Figure S1.** XRD patterns of the as-prepared precursors of ZCO, ZCO@C-2, ZCO@C-5, and ZCO@C-10.

**Figure S2.** XRD pattern of the ZCO@C-10.

**Figure S3.** FTIR spectra of ZCO, ZCO@C-2, ZCO@C-5, and ZCO@C-10.

**Figure S4.** Raman pattern of ZCO@C-5 composite.

**Figure S5.** XRD pattern of carbon derived from pure D-glucose treated by hydrothermal reaction and high temperature calcination.


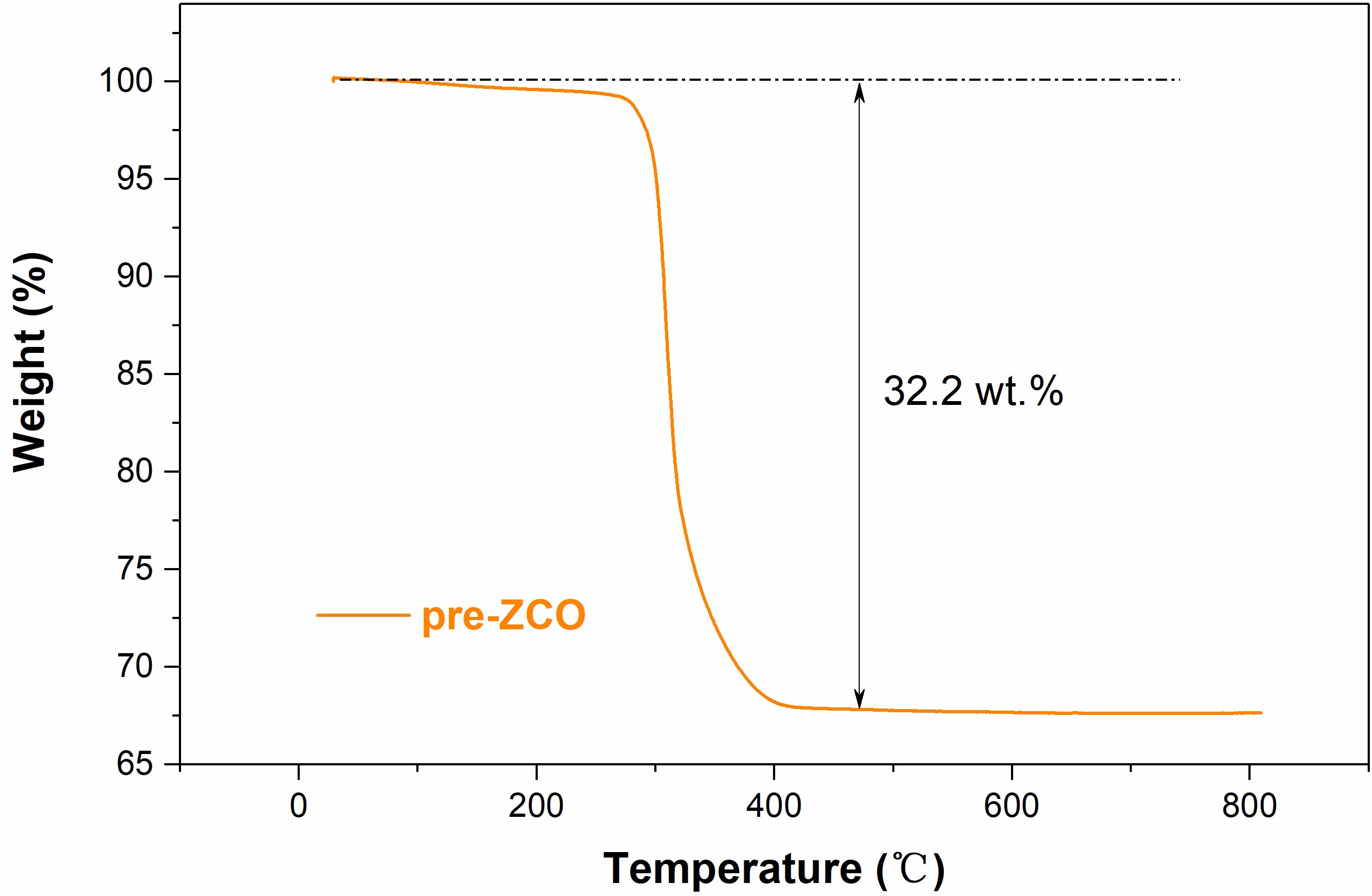


**Figure S6.** TG curve of the pure Zn-Co carbonate precursor.

ZnCo_2_(CO_3_)_3_→ZnCo_2_O_4_ + 3CO_2_↑ (Eqn. S1)

Figure S6 shows TG curve of the pure Zn-Co carbonate precursor. It presents a distinct weight loss step at about 320 °C, and the weight of annealing product hardly changes after 400 °C, indicating complete decomposition of the precursor from Zn-Co carbonate to Zn-Co oxide (Eqn. S1). And, the weight loss of 32.2% is close to the theoretical value (36.3%).

**Figure S7.** TG curves of the as-prepared (a) ZCO@C-2, (b) ZCO@C-5, and (c) ZCO@C-10 composites.

In Figure S7, the weight loss before 200 °C is mainly from the evaporation of the absorption water and the decomposition of residual oxygen-containing groups. Then, the further decreasing weight for the carbonaceous ZCO samples up to 700 °C can be assigned to the decomposition of amorphous carbon.


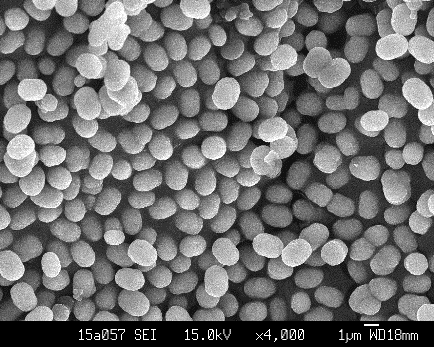

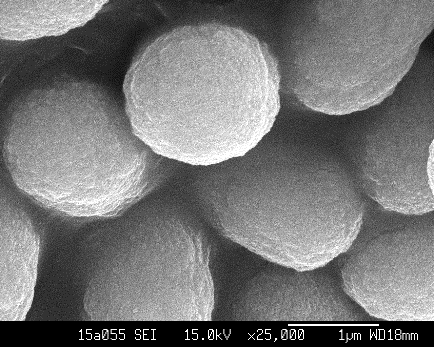


**Figure S8.** SEM images of ZCO@C-5 precursor.


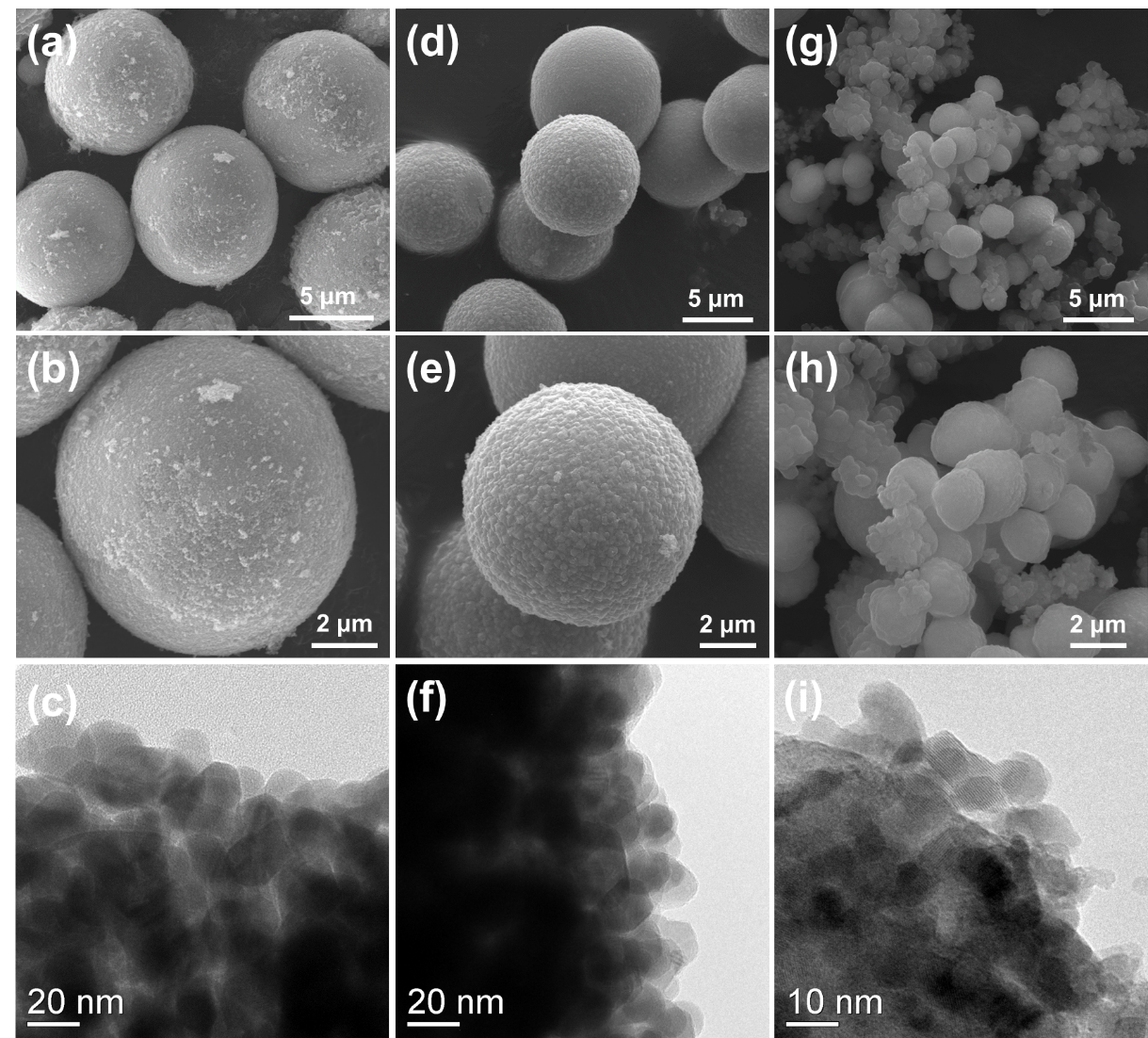


**Figure S9.** SEM and TEM images of (a-c) pure ZCO, (d-f) ZCO@C-2 and (g-i) ZCO@C-10 samples.

The sizes of small ZCO particles in ZCO, ZCO@C-2 and ZCO@C-10 samples were measured to be 21.2 nm, 20.5 nm, and 9.7 nm. ZCO@C-10 sample exhibit smaller ZCO particle, which is because the growth of metal ions was influenced by a large number of oxygen-containing functional groups of glucose molecules.


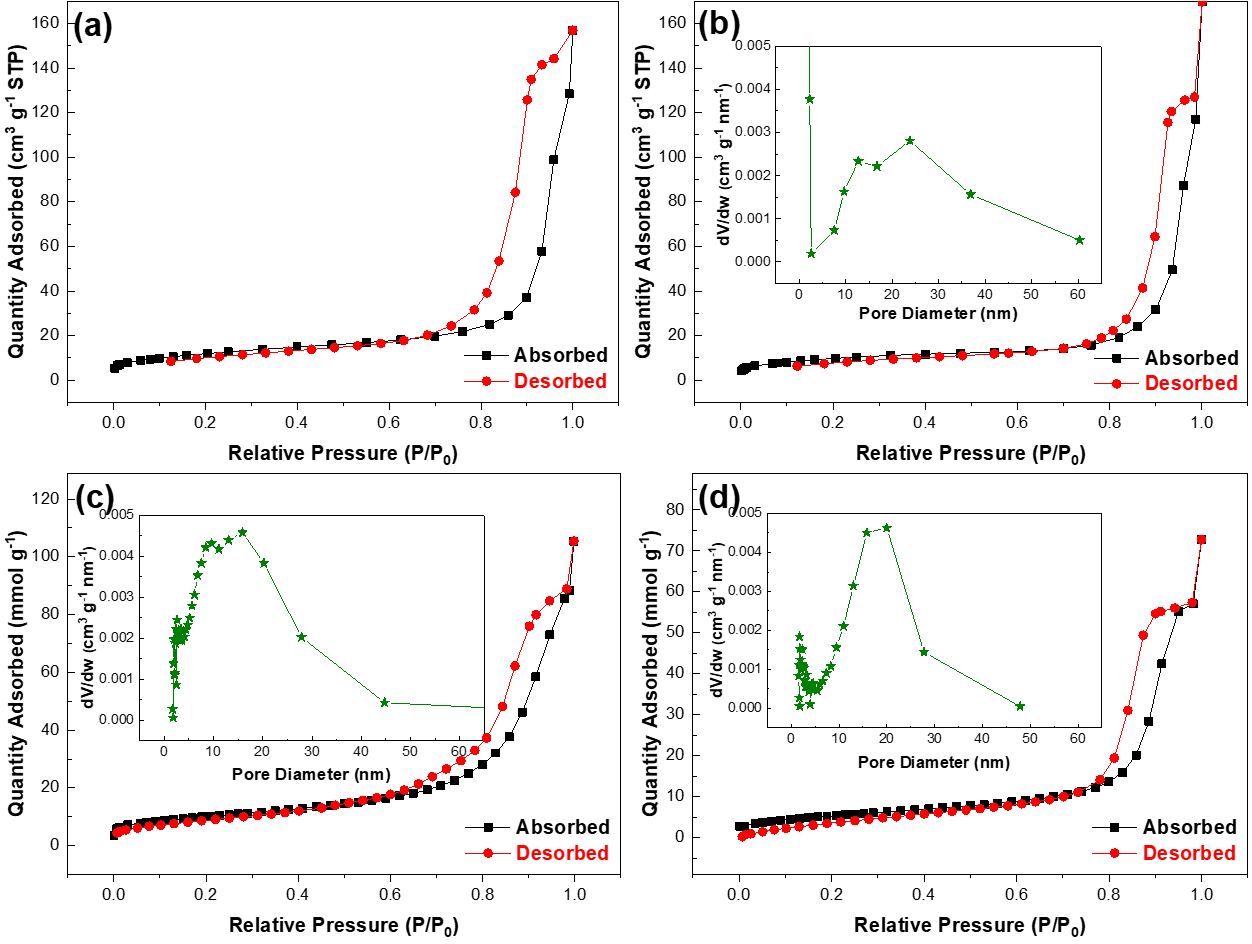


**Figure S10.** Nitrogen adsorption-desorption isotherm of ZCO@C-5 porous spheroids (a). Nitrogen adsorption-desorption isotherm and corresponding pore size distribution (inset) of (b) ZCO, (c) ZCO@C-2, and (d) ZCO@C-10.

**Figure S11.** Discharge-charge voltage profiles of pure ZCO and ZCO@C-5 composites at various current densities: 500 mA g^-1^, 1000 mA g^-1^, 2000 mA g^-1^, and 4000 mA g^-1^.


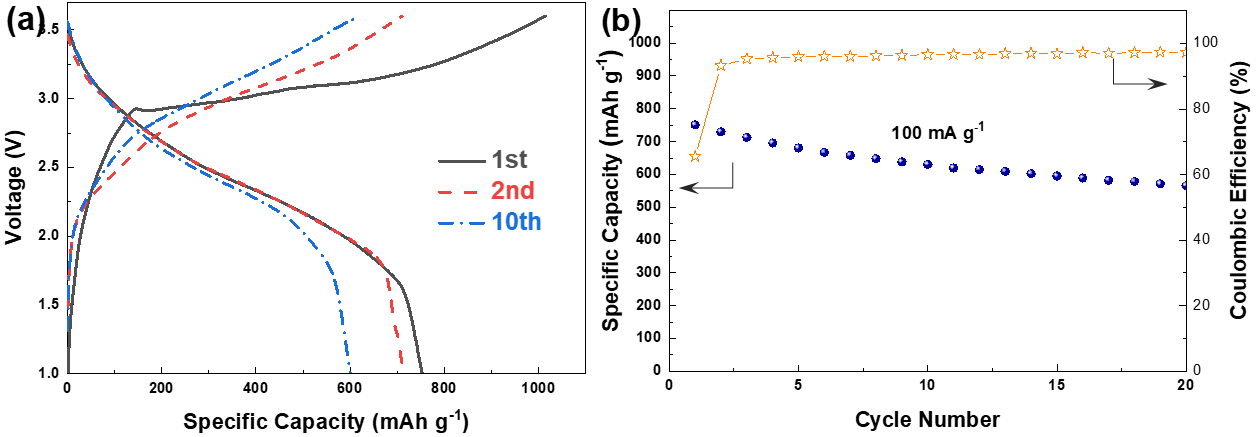


**Figure S12.** (a) Discharge-charge voltage profiles, and (b) cycling performance at 100 mA g^-1^ with corresponding coulombic efficiency of the LMO/ZCO@C-5 full cell.


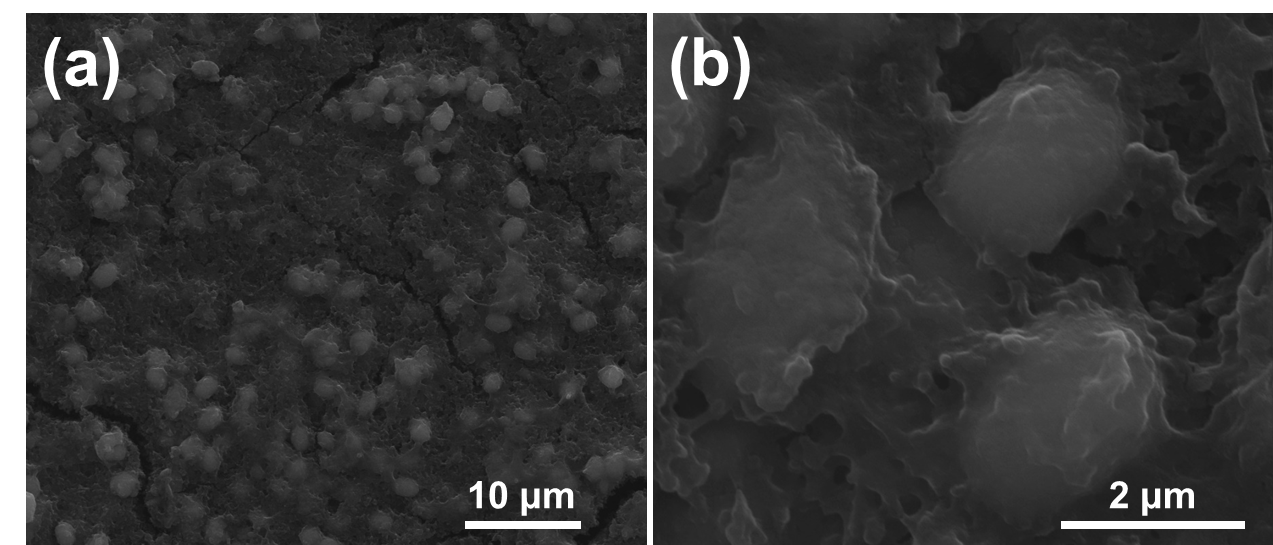


**Figure S13.** SEM images of ZCO@C-5 electrode after 600 cycles.

SEM images of ZCO@C-5 electrode after 600 cycles have been tested, as shown in Figure S13. After a long-term cycling, only a few cracks appear on the electrode surface and ellipsoidal structure of ZCO@C-5 is still well maintained.

**Table S1.** Zn and Co content of ZCO and ZCO@C-5 samples determined by ICP-MS.

|  | ZCO | ZCO@C-5 |
| --- | --- | --- |
| Zn (mg/L) | 0.496 | 0.547 |
| Co (mg/L) | 1.040 | 1.13 |
| Co/Zn (Atom) | 2.29 | 2.32 |

**Table S2.** Kinetic parameters of pure ZCO electrode and ZCO@C-5 composites electrode.

|  | R_e_  (Ω) | R_ct_  (Ω) | CPE  (S s^n^) | n  (0<n<1) | Z_w_  (S s^0.5^) | C_int_  (F) |
| --- | --- | --- | --- | --- | --- | --- |
| ZCO | 1.72 | 31.3 | 1.149×10^-5^ | 0.8374 | 0.0448 | 2.367×10^-3^ |
| ZCO@C-5 | 1.69 | 26.2 | 2.504×10^-5^ | 0.9136 | 0.1038 | 8.189×10^-4^ |

**Table S3.** Performance comparison between our materials with representative anode materials.

| Materials | Cycling performance | Rate capability | Ref. |
| --- | --- | --- | --- |
| Hollow octahedral ZnCo_2_O_4_ nanocages | 1025 mAh g^-1^ after 200 cycles at 500 mA g^-1^ | 525 mAh g^-1^ at 4000 mA g^-1^ | [1] |
| Sliced orange-shaped ZnCo_2_O_4_ | 600 mAh g^-1^ after 300 cycles at 1000 mA g^-1^ | 420 mAh g^-1^ at 5000 mA g^-1^ | [2] |
| Porous Te@ZnCo_2_O_4_ Nanofibers | 956 mAh g^-1^ after 100 cycles at 100 mA g^-1^ | 307 mAh g^-1^ at 2000 mA g^-1^ | [3] |
| Mesoporous rose-like ZnCo_2_O_4_ | 1000 mAh g^-1^ after 50 cycles at 100 mA g^-1^ | 800 mAh g^-1^ at 500 mA g^-1^ | [4] |
| CNTs anchored with Zn_x_Co_3-x_O_4_ nanocubes | 600 mAh g^-1^ after 300 cycles at 500 mA g^-1^ | 337 mAh g^-1^ at 1000 mA g^-1^ | [5] |
| Porous ZnCo_2_O_4_ decorated with rGO/CNTs | 728 mAh g^-1^ after 300 cycles at 1000 mA g^-1^ | 541 mAh g^-1^ at 4000 mA g^-1^ | [6] |
| ZCO microspheres | 631 mAh g^-1^ after 120 cycles at 500 mA g^-1^ | 407 mAh g^-1^ at 4000 mA g^-1^ | This work |
| Porous ZCO@C-5 spheroids | 815 mAh g^-1^ after 500 cycles at 2000 mA g^-1^ | 818 mAh g^-1^ at 4000 mA g^-1^ | This work |

**References**

[1] B.H. Liu, H. Liu, M.F. Liang, L.X. Liu, Z.L. Lv, H. Zhou, H. Guo, Controlled Synthesis of Hollow Octahedral ZnCo_2_O_4_ Nanocages Assembled from Ultrathin 2D Nanosheets for Enhanced Lithium Storage, Nanoscale, 9 (2017) 17174–17180.

[2] J.J. Deng, X.L. Yu, Y.B. He, B.H. Li, Q.H. Yang, F.Y. Kang, A Sliced Orange-Shaped ZnCo_2_O_4_ Material as Anode for High-Performance Lithium Ion Battery, Energy Storage Mater, 6 (2017) 61–69.

[3] G. Huang, Q. Li, D.M. Yin, L.M. Wang, Hierarchical Porous Te@ZnCo_2_O_4_ Nanofibers Derived from Te@Metal-Organic Frameworks for Superior Lithium Storage Capability, Adv. Funct. Mater., 27 (2017) 1604941.

[4] Y.J. Wang, J. Ke, Y.W. Zhang, Y.H. Huang, Microwave-assisted rapid synthesis of mesoporous nanostructured ZnCo_2_O_4_ anode materials for high-performance lithium-ion batteries, J. Mater. Chem. A, 3 (2015) 24303–24308.

[5] R. Chen, Yi Hu, Z. Shen, Y. Chen, X. He, X. Zhang, Y. Zhang, Controlled Synthesis of Carbon Nanofibers Anchored with ZnxCo_3–x_O_4_ Nanocubes as Binder-Free Anode Materials for Lithium-Ion Batteries, ACS Appl. Mater. Interfaces, 8 (2016) 2591–2599.

[6] Z. Wang, Q. Ru, Y.D. Mo, L.Y. Guo, X.Q. Chen, X.H. Hou, S.J. Hu, Facile synthesis of porous peanut-like ZnCo_2_O_4_ decorated with rGO/CNTs toward high-performance lithium ion batteries, J Mater Sci-Mater El, 28 (2017) 9081–9090.
